# Prevalence and risk factors associated with repeat breeding of beef cattle in Sleman Regency, Indonesia

**DOI:** 10.14202/vetworld.2022.870-877

**Published:** 2022-04-08

**Authors:** Rian Maulana, Heru Susetya, Surya Agus Prihatno

**Affiliations:** 1 Veterinary Science Postgraduate Program, Faculty of Veterinary Medicine, Universitas Gadjah Mada, Yogyakarta 55281, Indonesia; 2 Department of Veterinary Public Health, Faculty of Veterinary Medicine, Universitas Gadjah Mada, Yogyakarta 55281, Indonesia; 3 Department of Reproduction and Obstetrics, Faculty of Veterinary Medicine, Universitas Gadjah Mada, Yogyakarta 55281, Indonesia

**Keywords:** breeder, cow, epidemiology, reproductive failure, statistical analysis

## Abstract

**Background and Aim:**

Various management practices may cause the occurrence of reproductive failure indicated by repeat breeding in beef cattle. This study aimed to estimate the prevalence and the risk factors of repeat breeding in beef cattle in Sleman Regency, Indonesia.

**Materials and Methods:**

Observational and cross-sectional studies were used to determine the prevalence and the risk factors of repeat breeding. Sampling was conducted using a multistage cluster design. The sample size was determined using a sampling formula (n=4 PQ/L2). Questionnaire and interview data were evaluated descriptively. Chi-square analysis and odds ratio (OR) test were conducted to determine the association and association strength with a confidence level of 95%. Univariate, bivariate, and multivariate analysis through multivariate logistic regression test was done using Statistical Package for the Social Sciences, version 21.0 software.

**Results:**

The results indicated that the prevalence of repeat breeding in beef cattle in the Sleman Regency was 30.4%. Multivariate analysis indicated that risk factors that significantly affected the repeat breeding were breeding experience (p=0.000; OR=3.378), knowledge of estrus cycle (p=0.000; OR=5.263), feed type (p=0.001; OR=6.061), feeding frequency (p=0.003; OR=2.77), shed hygiene (p=0.33; OR=2,381), and drainage system (p=0.000; OR=4,484).

**Conclusion:**

Various husbandry management significantly influence the incidence of repeat breeding in beef cattle in Sleman Regency with the type of feed, which was the highest risk factor. Hay should not be used as the main feed source since it might increase the incidence of repeat breeding. However, the other environmental factors such as season and presence of infection or parasite also need to be investigated further.

## Introduction

Based on the data from the Central Bureau of Statistics of Indonesia, in 2020, the demand for beef meat was still higher than its availability in the market. The Indonesian government has launched policies to support the meat self-sufficiency program, but this has not been achieved until now. A problem in the field is that there are still many reproductive disorders in cows [1,2]. Reproductive disorders related to infertility, such as inactive ovaries, ovary hypofunction, ovarian cystic, and endometritis, cause low reproductive efficiency. In addition, repeat breeding is a subfertile condition that indicates reproductive disorders and decreases reproductive performance [3]. Repeat breeding is defined as a condition in which a cow fails to conceive after being bred 3 or more times with a fertile bull or inseminated using the semen of a fertile bull without any clinical complications observed [4].

The prevalence of repeat breeding in beef cattle varies among the regions of Indonesia. Juliana *et al*. [5] reported that the prevalence of repeat breeding in Bali cattle in the Pringsewu Regency was 19.85%. A similar result was reported by Subagio *et al*. [6] that the prevalence of beef cattle in the Jombang Regency was 21.12% and that it fluctuates every year. Differing from two regencies before, in the Banyuwangi Regency, the prevalence of repeat breeding is very high and reaches 64% [7], while Nasution *et al*. [8] reported that in the North Labuhanbatu Regency, the prevalence of repeat breeding in beef cattle was 17.3%. Several factors are associated with the repeat breeding of beef cattle. The failure of fertilization and embryonic death are the two leading causes of repeat breeding in cows. However, the factors related to reproductive failure come from the cows and bulls as well as the management [9]. Good management strategies are needed to increase the reproductive performance of beef cattle [10].

Good management practices are essential for the successful breeding of beef cattle. However, various levels of knowledge and educational background can affect management practice. In Sleman Regency, Indonesia, the breeders have diverse experiences and educational backgrounds that result in the various practices of management husbandry of beef cattle in small herds. Different management practices may lead to reproductive failure shown by repeat breeding.

This study aimed to estimate the prevalence and the risk factors of repeat breeding in small herds located in Sleman Regency, Indonesia. The knowledge of risk factors can be used to reduce the future occurrence of repeat breading.

## Materials and Methods

### Ethical approval

This study was approved (No. 00036/EC-FKH/Int./2021) by the research committee of the Faculty of Veterinary Medicine, Universitas Gadjah Mada.

### Study period and location

The study was conducted from January to March 2021. There are 17 districts in the Sleman Regency. Eight districts, namely, Seyegan, Gamping, Berbah, Prambanan, Ngemplak, Ngaglik, Sleman, and Pakem districts, were chosen as sampling locations based on the multistage cluster sampling method.

### General husbandry practices in Sleman Regency

During the study period, Indonesia undergoes the rainy season in all regions. The average daily temperature in Sleman Regency was 26.1°C with the maximum temperature was 31.3°C and the minimum temperature was 22.8°C. The average humidity was 84%, with the maximum humidity was 93% and the minimum humidity was 93%. The altitude of the Sleman Regency area ranges from 79 to 634 masl. Almost half of the area is fertile agricultural land supported by technical irrigation in the west and south. Thus, agricultural waste such as hay is abundant in the Sleman Regency. Commonly the farmer utilizes this agricultural waste as cattle feed. The other main cattle feed is grass forages. The feeding was combined with feed concentrates such as mixed rice bran and mineral supplements. Beef cattle management is carried out with an intensive system in the individual or herd housing system. Artificial insemination is used as a breeding system. Estrus detection is carried out using direct observation by the farmer.

### Study design

Several livestock groups from eight districts in the Sleman Regency, Indonesia, were included in this research. Data identification and analysis were conducted at the Faculty of Veterinary Medicine, Universitas Gadjah Mada, Yogyakarta. A cross-sectional study with a multistage cluster sampling method was used in this research. The criteria of the samples were breeders who have beef cattle that have calved at least once, aged 3-8 years, have normal estrus cycle, are in healthy condition, and are traditionally managed by either individuals or in groups. The sample size was determined based on the beef cow population because of the lack of data on the exact number of breeders in the Sleman Regency. The number of cattle samples was determined using the formula n=4 PQ/L² (n: The size of the cattle sample, P: Assumed incidence rate in the study area (19.85% due to Juliana *et al*. [5]), Q: (1-P), L²: Desired confidence level [95%]). The sample size was multiplied by five (n=225×5=1275 cows) to avoid bias. Assuming a breeder has three cows, the sample size of the breeder was determined to be as much as 425 people (n=1275:3). The data include the number of cows with repeat breeding, education background of the breeder, breeding experience, knowledge about estrus cycle and estrus signs, the number of estrus observations, breeding time, drinking water and water sources, feed and feeding frequency, type of shed floor, shed hygiene, drainage system, and body condition scoring (BCS) were recorded for descriptive and statistical analyses.

### Data collection

Data on the beef cattle population were obtained from the Department of Agriculture, Food and Fisheries, Sleman Regency. Primary data on the number of cows with repeat breeding, education background of the breeder, breeding experience, knowledge on estrus cycle and estrus sign, the number of observations, breeding time, drinking water and water sources, feed and feeding frequency, type of shed floor, shed hygiene, drainage system, and BCS were acquired by direct interviews with the breeder and direct observations in the farm. Information was obtained from the interview, and the observation was recorded in Excel form (Microsoft Excel 365^®^).

### Statistical analysis

Statistical analysis was conducted using the Statistical Packages for the Social Science program version 21.0 (IBM Corp., NY, USA). Descriptive data on the prevalence and risk factors of repeat breeding were shown. Univariate analysis was conducted to calculate the frequency of repeat breeding (dependent variable) and the frequency of each risk factor (the independent variable). Bivariate analysis was conducted to determine the relationship between the independent and dependent variables. The Chi-square test determined the independent variables that meet the criteria for the basic model (p<0.25) with a 95% confidence level and 5% desired error. An odds ratio (OR) test was conducted when there was a significant relationship between the risk factor and the incidence of repeat breeding to observe the association strength. The first step of the analysis was to develop the basic model of bivariate analysis. Variables that did not meet p<0.05 were gradually removed, starting from the largest p-value. A confounding test was performed during this step to determine if the excluded variable could change OR >10%. It is called a confounding variable, and this variable was re-entered into the model because it was an essential variable. Whether the change is OR <10%, the variable was permanently excluded and considered an unimportant variable or had no effect. The final step of the multivariate analysis was conducting moderated regression analysis to verify the final modeling.

## Results

Four hundred and twenty-five breeders were included as respondents in this study. The Prambanan district had the highest proportion of respondents, with 160 breeders. The smallest respondents’ proportion was in Gamping and Pakem district, with 22 respondents. The prevalence of repeat breeding in each district is indicated in Table-1. The frequency of repeat breeding cases (dependent variable) and the frequency of risk factors for each category (independent variable) are shown in Tables-2 and 3. This study showed that the prevalence of repeat breeding in beef cattle in the Sleman Regency was 30.4%. Bivariate analysis is also known as the Chi-square test, using a 2×2 table (dichotomy) showed the relationship between the dependent and independent variables. Many variables, such as education background, breeding experience, estrus observations, water sources, and type of shed floor, were merged into dichotomy data to perform the Chi-square test. The results indicated that some factors, including last education background, estrus observation, breeding experience, knowledge of estrus cycle, feed, feeding frequency, shed hygiene, and drainage system, have a significant relationship with the occurrence of repeat breeding, while factors, including knowledge of estrus signs, drink, water source, and type of shed floor, have no significant relationship with the occurrence of repeat breeding (Table-4). Final multivariate analysis indicated six risk factors, including breeding experience, knowledge of estrus cycle, feed, feeding frequency, shed hygiene, and drainage system, that truly have a significant relationship with the occurrence of repeat breeding (Table-5). Two variables, including estrus observation and last education background, were excluded due to a change in OR<10%. Shed hygiene was a risk factor with the weakest relationship with repeat breeding, while feed was a risk factor with the strongest relationship with repeat breeding (Table-6).

**Table 1:** The prevalence of repeat breeding in beef cattle in Sleman Regency, Indonesia.

No.	Districts	Number of samples	Number of cases	Prevalence (%)
1	Seyegan	36	10	27.8
2	Gamping	22	4	18.2
3	Berbah	48	14	29.2
4	Prambanan	160	55	34.4
5	Ngemplak	53	23	43.4
6	Ngaglik	42	11	26.2
7	Sleman	42	8	19.0
8	Pakem	22	4	18.2
	Sleman Regency	425	129	30.4

**Table 2:** Descriptive data of risk factors for repeat breeding in beef cattle in Sleman Regency, Indonesia.

Variable	Category	Number of samples		Average

		(n)	(%)	
Occurrence of repeat breeding	Yes	129	30.4	-
	No	296	69.6	
Education background	No	14	3.3	-
	Elementary school	98	23.1	
	Junior high school	97	22.8	
	Senior high school	206	48.5	
	Academy	1	0.2	
	Bachelor	9	2.1	
Breeding experience (year)	1-5	38	8.9	14.7
	6-10	94	22.1	
	11-15	77	18.1	
	16-20	118	27.8	
	>20	98	23.1	
Knowledge of estrus cycle	Do not know	97	22.8	-
	Know	328	77.2	
Knowledge of estrus signs	Do not know	8	1.9	-
	Know	417	98.1	
Estrus observation (times per day)	No observation	0	0	2-3
	1	7	1.6	
	2	284	66.8	
	3	127	29.9	
	4	7	1.6	
Breeding time (h after show estrus signs)	0-4	30	7.1	9.9
	5-8	32	7.5	
	9-12	328	77.2	
	>12	35	8.2	
Drink	Libitum	423	99.5	-
	*Ad libitum*	2	0.5	
Water source	River	2	0.5	-
	Well	423	99.5	
	Rainwater storage	0	0	
Feed	More hay	43	10.1	-
	More forage	382	89.9	
Feeding frequency (times per day)	1-2	296	69.6	-
	3-4	129	30.4	
Type of shed floor	Soil	4	0.9	-
	Cement	421	99.1	
	Rubber	0	0	
Shed hygiene	Poor	90	21.2	-
	Good	335	78.8	
Drainage system	Poor	173	59.3	-
	Good	252	40.7	
BCS	1.5-2.0	55	12.9	2.6
	2.5-3.0	360	84.7	
	3.5-4.0	10	2.4	

**Table 3:** Data distribution of repeat breeding risk factors for breeding time and body condition scoring of beef cattle in Sleman Regency, Indonesia.

Variable	Category	Repeat breeding		n	Average

		Yes (%)	No (%)		
Breeding time: (h after show estrus signs)	0-4	18 (60)	12 (40)	30	9.9
	5-8	26 (81.3)	6 (18.8)	32	
	9-12	61 (18.6)	267 (81.4)	328	
	>12	24 (68.6)	11 (31.4)	35	
BCS	1.5-2.0	38 (69.1)	17 (30.9)	55	2.6
	2.5-3.0	91 (25.3)	269 (74.7)	360	
	3.5-4.0	0 (0)	10 (100)	10	

**Table 4:** Bivariate analysis of risk factors for repeat breeding in beef cattle in Sleman Regency, Indonesia.

Variable	Repeat breeding		n	p-value	OR
	Yes (%)	No (%)			
Knowledge of estrus signs				0.999	-
Do not know	8 (100)	0 (0)	8		
Know	121 (29)	296 (71)	417		
Drink				1.000	-
Libitum	129 (30.5)	294 (69.5)	423		
*Ad libitum*	0 (0)	2 (100)	2		
Water source				0.515	2.305
River	1 (50)	1 (50)	2		
Well	128 (30.3)	295 (69.7)	423		
Type of shed floor				0.588	2.315
Soil	2 (50)	2 (50)	4		
Cement	127 (30.2)	294 (69.8)	421		
Last education background				0.000	0.170
<Senior high school	88 (42.1)	121 (57.9)	209		
≥Senior high school	175 (81)	41 (19)	216		
Estrus observation (times per day)				0.002	2.154
1-2	102 (35)	189 (65)	291		
3-4	27 (20)	107 (80)	134		
Breeding experience (years)				0.000	2.779
1-15	85 (40.7)	124 (59.3)	209		
≥16	44 (20.4)	172 (79.6)	216		
Knowledge of estrus cycle				0.000	8.955
Do not know	66 (68)	31 (32)	97		
Know	63 (19.2)	265 (80.8)	328		
Feed				0.000	6.579
More hay	30 (69.8)	13 (30.2)	43		
More forage	99 (25.9)	283 (74.1)	382		
Feeding frequency (times per day)				0.000	2.571
1-2	106 (35.8)	190 (64.2)	296		
>2	23 (17.8)	106 (82.2)	129		
Shed hygiene				0.000	6.739
Poor	58 (64.4)	32 (35.6)	90		
Good	71 (21.2)	264 (78.8)	335		
Drainage system				0.000	7.377
Poor	94 (54.3)	79 (45.7)	173		
Good	35 (13.9)	217 (86.1)	252		

OR=Odds ratio

**Table 5:** Bivariate analysis between the frequency of repeat breeding and the risk factors of repeat breeding in beef cattle in Sleman Regency, Indonesia.

Variable	p-value	Decision
Last education background	0.000	Possibly included
Breeding experience	0.000	Possibly included
Knowledge of estrus cycle	0.000	Possibly included
Knowledge of estrus signs	0.999	Excluded
Estrus observation	0.002	Possibly included
Drink	1.000	Excluded
Water source	0.515	Excluded
Feed	0.000	Possibly included
Feeding frequency	0.000	Possibly included
Type of shed floor	0.588	Excluded
Shed hygiene	0.000	Possibly included
Drainage system	0.000	Possibly included

**Table 6:** Multiple logistics regression analysis of risk factors for repeat breeding in beef cattle in Sleman Regency, Indonesia.

Variable	Coeff. B	p-value	OR	95% CI
Breeding experience	1.217	0.000	3.378	1.767-4.452
Knowledge of estrus cycle	1.662	0.000	5.263	4.695-7.309
Feed	1.800	0.001	6.061	2.160-8.949
Feeding frequency	1.019	0.003	2.770	1.412-4.435
Shed hygiene	0.869	0.033	2.381	1.075-4.291
Drainage system	1.500	0.000	4.484	2.179-7.259

OD=Odds ratio, CI=Confidence interval

## Discussion

The prevalence of repeat breeding in beef cattle in the Sleman Regency differs from the other reports in other regions in Indonesia [5-8]. This might be caused by various husbandry systems in each region. In Sleman Regency, the primary husbandry system is intensive, with artificial insemination as a breeding system. In other regions, such as the Mina Regency of Southeast Sulawesi Province, the main husbandry system is not intensive but semi-intensive and extensive with the natural breeding system [11]. According to Gupta and Deopurkar, the prevalence of repeat breeding differs among locations in the world, ranging from 5% to 32% [12]. Moreover, in a tropical environment, the prevalence of repeat breeding can reach 62% [13]. Based on the district area, the highest prevalence of repeat breeding in beef cattle was found in Ngemplak district with 43.4%, while the lowest prevalence was found in Pakem and Gamping district with 18.2%. From the direct observation of farms in Ngemplak district, many sheds in poor condition and hay were found as the primary feed.

In reproductive management, breeding time is crucial for breeding using artificial insemination. Almost all respondents used artificial insemination for their breeding system. Most breeders (77.2%) bred their cow 9-12 h after showing estrus signs. This category of breeding time has the lowest repeat breeding cases (18.6%). This agrees with Diskin [14], who reported a non-return rate reaching 60.7% when artificial insemination is performed 6-12 h after detecting estrus signs. According to Perez-Marin *et al*. [15], the best time for artificial insemination is using the am/pm system, where if the start of estrus occurs at night, then insemination is performed in the morning of the next day. Too early or too late bred can cause pregnancy failure characterized by repeat breeding [16].

This study used a scale for BCS from 0 to 5 [17]. Most beef cattle in the Sleman Regency (84.7%) have BCS ranging from 2.5 to 3.0. However, the highest repeat breeding case was in the beef cattle group with BCS ranging from 1.5 to 2.0 as much as 69.1%. According to Stevenson *et al*. [18], cows with a BCS of <2.25 had lower progesterone concentrations than cows with a higher BCS. Squires also stated that poor nutrition reduced plasma insulin, insulin-like growth factor-I (IGF-I), and leptin levels and increased growth hormone [19]. IGF-I cooperates with gonadotropins to stimulate follicular development, and low levels of IGF-I in follicular fluid are associated with low ovulation rates.

It was revealed that knowledge of estrus signs has an insignificant relationship with the occurrence of repeat breeding, while the knowledge of the estrus cycle and the number of estrus observations have a significant relationship with the event of repeat breeding. Cows belonging to the breeders who do not know the estrus cycle will experience repeat breeding 5.263 times higher than cows belonging to the breeders who know the estrus cycle. The results of this study agree witha report by Subagyo [20], who stated that cows belonging to the breeders who did not know the estrus cycle have a chance to experience repeat breeding 2.3 times higher than cows belonging to the breeders who know the estrus cycle. In addition, a small number of estrus observations (1-2 times/day) have a higher percentage of repeat breeding than more estrus observations (3-4 times/day). Most breeders (66.8%) only made estrus observations twice a day. According to Prihatno *et al*. [21], ideally, breeders should observe estrus signs 4 times a day, in the morning, afternoon, evening, and night, with an observation period of about 5-10 min. Poor estrus detection contributes greatly to low fertility [14]. Errors in detecting estrus can cause the failure of the artificial insemination program [22].

The Chi-square test showed no significant relationship between drink *libitum* or *ad libitum* and the occurrence of repeat breeding. Most of the breeders (99.5%) give *libitum* drink water, and only 0.5% of the breeders give *ad libitum* drink water to their cattle. Drinking water is usually obtained from wells (99.5%) and rivers (0.5%). The source of drinking water also has no significant relationship with the occurrence of repeat breeding. Water is essential for maintaining body fluids, ionic balance, digestion, absorption, nutrient metabolism, removing unnecessary substances, reducing excess heat, maintaining the fetal fluid environment, and transporting nutrients to or from body tissues [23]. If these functions are disturbed, it will also affect the cow’s reproductive system. The drinking water provided must be clean, fresh, clear, and does not contain harmful microorganisms [24]. It is not recommended to obtain drinking water from the river. Although cattle do not exhibit any disease symptoms when drinking water from the river, but they are more likely to get sick than cows that drink water from wells or groundwater [25].

Slippery and uneven floors can cause lameness, and a muddy floor can cause foot rot in cows [26]. Diseases induced by bad flooring will cause stress to the cow. Perez-Marin *et al*. [15] reported that stress induction will increase the hormone cortisol, thereby decreasing progesterone levels. Thus, stress can be considered a potential factor in the incidence of repeat breeding. Ferreira *et al*. [27] also reported that stress in cattle would decrease the duration and estrus intensity. In this study, the type of shed floor did not significantly correlate with the occurrence of repeat breeding.

The final multivariate analysis excluded the last education background as a risk factor for repeat breeding of beef cattle in the Sleman Regency. This agrees with that of Prihatno *et al*. [21], who stated that education background is not directly proportional to the incidence of repeat breeding. However, according to Nath *et al*. [28], higher experience in animal husbandry can help breeders in overcoming the challenges that arise when managing the farm. This agrees with our result that breeding experience has a significant relationship with the occurrence of repeat breeding. The cows belonging to the breeders with 1-15 years of breeding experience can experience repeat breeding 3.378 times higher than cows belonging to the breeders who have more than 15 years of breeding experience. There are several vital things that breeders should know to decrease the incidence of repeat breeding, including estrus detection, insemination time, cow nutrition management, and other knowledge that can affect the condition of their cows [29].

Based on the results of this study, the feed was one of the highest risk factors for repeat breeding in beef cattle. The cows belonging to the breeders whose cows were fed more often using hay than forage will possibly experience repeat breeding 6.061 times higher. Celik *et al*. [30] stated that β-carotene contained in the feed would increase the corpus luteum and follicle size function, causing high levels of estradiol and progesterone, which helps increase the pregnancy rate by 33% compared to the control group (27%). Pradhan and Nakagoshi [31] reported that when the cows lack carbohydrates and protein intake, the follicles will fail to develop, and follicular atresia will occur, characterized by anestrus. The hay has the features of low crude protein content (2-5%) and high crude fiber, including cellulose, hemicellulose, lignin, and silica [32]. Meanwhile, forage and legumes exhibited much higher crude protein content compared to hay. An example of the type *Panicum maximum* has crude protein content of 12.75% [33] and crude protein content of *Leucaena leucocephala* is 24% [34]. The crude protein needed for cattle is at least 12% [35]. This shows that the nutritional value of hay could not fulfill the cows’ needs. The same thing was reported by Yanuartono *et al*. [36] that hay does not contain enough glucose, amino acids, and minerals for microbial growth in the rumen. It has been known for a long time that 80% of the variance in fertility is due to environmental factors, of which more than 50% is explained by nutrition when severe infections and male fertility are excluded [37]. In addition, feeding frequency also has a significant relationship with the occurrence of repeat breeding. Cows fed 1-2 times a day can experience repeat breeding 2.77 times higher than cows fed 3-4 times a day. However, the ideal management of feeding is to limit the amount of feed but with sufficient quality and quantity [35].

Shed hygiene and the availability of drainage systems were risk factors that have a significant relationship with the occurrence of repeat breeding. Cows that were maintained in poor shed hygiene and did not have a drainage system in the shed can experience repeat breeding 2.381 and 4.484 times higher than cows that were maintained in good hygiene and had a drainage system in the shed. According to Janowski *et al*. [38], good hygiene of the shed and cows is required to avoid reproductive disorders. Livestock waste generated from livestock activities in the form of leftover feed, urine, while feces contain methane gas (CH_4_) and nitrogen oxides (N_2_O) and contain microorganisms both pathogenic and non-pathogenic such as *Escherichia coli*, *Salmonella* spp., and *Shigella* spp. in the cage environment [39]. The accumulation of feces and urine in the shed may cause the cow lying down to become infected with bacteria in the uterine canal through the vulva as well as during or after insemination and may cause endometritis [21]. Endometritis can cause embryonic death presented by repeat breeding [15]. Sheldon *et al*. [40] reported that subclinical endometritis often occurs during the postpartum period and insemination treatment because at that time, the cervix opens until the involution process is complete, and this is supported by the conditions of the dirty shed environment. According to Fawaid, the cowshed needs to be equipped using a feed and sewage channel in front of the cowshed in the form of a small ditch that extends at the back of the cow’s position [39]. This channel allows the rest of the urine and feces to flow into the drainage system to always clean the shed.

## Conclusion

The prevalence of repeat breeding in beef cattle in the Sleman Regency, Indonesia, was 30.4%. The risk factors from husbandry management that have a significant influence on the incidence of repeat breeding in beef cattle in Sleman Regency were breeding experience, knowledge of estrus cycle, feed, feeding frequency, shed hygiene, and the availability of drainage system. Feeding with hay was the highest risk factor for repeat breeding in beef cattle in the Sleman Regency. However, the other environmental factors such as season and presence of infection or parasite also need to be investigated further.

## Authors’ Contributions

SAP: Conceptualization, supervision, formal analysis, project administration, and drafted and revised the manuscript. RM: Methodology, investigation, data curation, analysis, and the first draft of the manuscript. HS: Supervision, validation, and methodology. All authors read and approved the final manuscript.
